# Youth-friendly HIV self-testing: Acceptability of campus-based oral HIV self-testing among young adult students in Zimbabwe

**DOI:** 10.1371/journal.pone.0253745

**Published:** 2021-06-29

**Authors:** Andrea L. Koris, Kearsley A. Stewart, Tiarney D. Ritchwood, Daniel Mususa, Getrude Ncube, Rashida A. Ferrand, Grace McHugh

**Affiliations:** 1 Duke Global Health Institute, Duke University, Durham, NC, United States of America; 2 Family Medicine and Community Health, Duke University, Durham, NC, United States of America; 3 Biomedical Research and Training Institute, Harare, Zimbabwe; 4 Ministry of Health and Child Care, Harare, Zimbabwe; 5 Clinical Research Department, London School of Hygiene and Tropical Medicine, London, United Kingdom; University of the Witwatersrand, SOUTH AFRICA

## Abstract

**Background:**

Targeted HIV testing strategies are needed to reach remaining undiagnosed people living with HIV and achieve the UNAIDS’ 95-95-95 goals for 2030. HIV self-testing (HIVST) can increase uptake of HIV testing among young people, but user perspectives on novel distribution methods are uncertain. We assess the acceptability, perceived challenges, and recommendations of young adult lay counselor-led campus-based HIVST delivery among tertiary school students aged 18–24 years in Zimbabwe.

**Methods:**

We purposively sampled participants from an intervention involving campus-based HIVST using lay workers for distribution. We conducted in-depth interviews (IDIs) and focus group discussions (FGDs) among young adults from 10 universities and colleges in Zimbabwe who: (1) self-tested on campus; (2) self-tested off campus; and (3) opted not to self-test. We audio recorded and transcribed all interviews. Using applied thematic analysis, two investigators identified emergent themes and independently coded transcripts, achieving high inter-coder agreement.

**Results:**

Of the 52 young adults (53.8% male, 46.1% female) interviewed through 26 IDIs and four FGDs, most IDI participants (19/26, 73%) favored campus-based HIVST, describing it as a more autonomous, convenient, and socially acceptable experience than other facility or community-based HIV testing services. Despite general acceptability, participants identified challenges with this delivery model, including: perceived social coercion, insufficient privacy and access to post-test counseling. These challenges influenced some participants to opt against self-testing (6/52, 11.5%). Recommendations for improved implementation included integrating secondary distribution of test kits and increased HIV counseling options into campus-based programs.

**Conclusions:**

Barriers to HIV testing among young people are numerous and complex. As the number of new HIV infections among youth continue to grow worldwide, targeted strategies and youth friendly approaches that increase access to testing are needed to close the diagnostic coverage gap. This is the first study to describe young adult acceptance of campus-based delivery of HIVST by lay counselors in Zimbabwe.

## Introduction

HIV testing is a crucial entry point for uptake of HIV prevention and treatment services [1]. To meet the UNAIDS’ 95-95-95 targets for ending AIDS by 2030, 95% of people living with HIV (PLWHV) should be diagnosed, 95% of those diagnosed should be taking antiretroviral therapy (ART), and 95% of those on ART should be virally suppressed. Yet two decades of global health programming that expanded HIV testing at community- and facility-based sites achieved only 79% global diagnostic coverage, according to UNAIDS 2019 estimates [2]. This gap in diagnostic coverage reflects considerable heterogeneity across age group and geographic region. Youth (ages 15–24) are less likely to access HIV testing than adults (ages 25–49) [3], and they are disproportionately affected by HIV; in 2018, an estimated one third of all new infections occurred in the 15–24 age group [4], with 73% of new infections among youth occurring in Africa [5]. HIV self-testing (HIVST) may increase the uptake and frequency of testing for youth and others unlikely to test [6–8], and has shown to be an acceptable method to learn one’s HIV status without risk of self or social harm [9].

In Zimbabwe, despite strong gains made to increase diagnostic coverage over the last decade, approximately half of youth had tested for HIV according to a 2015–2016 Zimbabwe population-based survey (ZIMPHIA) [10]. Young people do not access HIV testing due to perceived or actual barriers of accessibility, perceptions of limited support from social networks, concerns over confidentiality and fear of stigma from healthcare providers [11–13]. They want to see convenient, non-judgmental, free HIV testing services prioritized and made available in spaces easily accessible to them [14, 15]. Youth-friendly delivery options for HIV testing are needed to improve knowledge of serostatus and reduce new infections among youth [16].

In 2015, Zimbabwe developed a national policy on HIVST and initiated large-scale implementation under the HIV Self-Testing Africa (STAR) initiative. Results from the STAR trial demonstrate how targeted roll-out of HIVST across multiple distribution models can increase coverage of HIV testing and contribute to case finding among populations unlikely to test, including youth [6]. While HIVST is available in a number of private clinics and pharmacies across Zimbabwe, offering HIVST and counseling in venues primarily frequented by youth may provide more accessible, less stigmatizing opportunities for them to test.

Delivery of HIVST on tertiary school campuses is one such option. In South Africa, where similarly to Zimbabwe youth account for a disproportionate number of new HIV infections but are less likely to test than adults [4, 17], a study investigating the acceptability of HIVST distribution on college campuses found the delivery of unsupervised HIVST was highly accepted by students [18]. A similar study in the Democratic Republic of Congo found that both supervised and unsupervised HIVST was favored among university students [19]. More research is needed to understand the factors that impact acceptability and uptake of HIVST at tertiary-level schools, so youth-focused testing strategies can be improved. Here, we describe Zimbabwean young adults’ (ages 18–24) [20] acceptability of a lay counselor-led campus-based distribution of HIVST, and their recommendations for future implementation.

## Methods

We conducted qualitative research within the Feasibility and Acceptability of HIV Self-Testing study (FAST), an interventional study that evaluated HIVST uptake among young adults at tertiary educational institutions in Zimbabwe. The qualitative sub-study was implemented concurrently with the FAST study, and it was conducted in accordance with the consolidated criteria for reporting qualitative research (see S1 File) [21].

### HIV testing methods

From June 2019 to March 2020, the FAST study distributed oral HIVST kits (OraQuick Rapid HIV-1/2 Anti body self-test®) for free to 6,296 young adults at 13 universities and technical vocational colleges across Zimbabwe. Prior to beginning distribution at each site, members of the study team advertised the program on campus radio shows and distributed brochures to generate traffic to the testing booths. Testing booths were staffed by trained young adult counselors who instructed participants to accurately use the oral swab and interpret test results. Participants elected to test on-site, or off-site at a location of their choosing. Participants who self-reported reactive test results received confirmatory blood-based testing by an on-site provider with the study team, in line with Zimbabwe National HIV testing policy [22]. Each person with a reactive test took two point of care rapid diagnostic test kits, one Standard Q HIV 1/2 Ab 4-Line® and one Chembio HIV 1/2 STAT-PAK®, which were administered in a private booth by one of the study team members certified in HIV testing and counseling. Participants who received a positive blood-based result received counselling and referrals to nearby clinics and had a follow up phone call within four weeks to determine if they had successfully linked to care. Participants eligible for the qualitative interviews and focus groups were students who (1) had not had an HIV test in the previous three months; (2) were not known to be living with HIV; and (3) tested on-site, off-site, or opted not to self-test when they approached the distribution points.

### Participants and recruitment

The qualitative research team consisted of lead qualitative researcher (ALK), research assistant (DM), and the FAST principal investigator (GM). In total, we recruited 52 young adults from 10 FAST distribution sites in Harare, Bindura, and Masvingo, Zimbabwe (see S1 Table). For IDIs, 26 individuals were purposively sampled from a list of participants who approached the distribution table to learn more about the study, opted in or out of HIVST, and consented to undertake a qualitative interview. We used a maximum variation approach [23] and intentionally varied the demographic attributes of participants by gender and testing selection (self-tested on-site, off-site, or opted not to test) in order to maximize a broad representation of participant types within each selected category (see Table 1).

**Table 1:** Purposive sampling framework of IDI participants.

Sex	HIVST	Number Recruited
	Selection	
Male	Tested on-site	4
	Tested off-site	3
	Opted not to test	4
Female	Tested on-site	6
	Tested off-site	7
	Opted not to test	2

We conveniently sampled 26 participants to participate in four FGDs. DM approached potential participants at the distribution sites; if they met the participant eligibility criteria and provided informed consent, they were scheduled to participate in a mixed-gender FGD.

### Data collection and qualitative analysis

All interviews were conducted by DM, a young adult Zimbabwean male research assistant trained in qualitative methods. He facilitated IDIs and FGDs in English, Shona or a mix of both, following the preference of the participant. Participants received 5 USD in airtime vouchers as compensation for their time. An additional member of the study team who was trained in youth-friendly qualitative data collection methods attended all FGDs to take notes and document nonverbal expressions of the participants. The notetaker, DM, and ALK convened after each FGD to discuss emergent themes and suggested probes for future FGDs. Audio recordings were transcribed in the language in which they were performed and translated into English, with key identifying information redacted. Another Shona speaking study member not involved in the facilitation, translation or transcription of the interviews reviewed translated transcripts against audio files, to optimize accuracy of translation. Interviews took place in a private location on campus, and lasted approximately 50 minutes. The pre-tested IDI topic guides contained open-ended probes related to acceptability and perceived challenges associated with campus-based HIVST (see S2 File). The FGD topic guide included open-ended questions that solicited young adults’ recommendations for improving youth-friendly delivery of HIVST at tertiary level schools (see S3 File). Data saturation was anticipated after approximately 25 interviews [24] and five FGDs [25]. After conducting qualitative activities at the first 10 FAST distribution sites, we reached thematic saturation.

We used applied thematic analysis to analyze data in NVivo 12 [26]. To create the codebook, we compared transcripts and grouped common responses under deductive codes derived from the topic guides, and inductive codes that emerged from the data. To pilot the preliminary codebook, ALK and DM independently coded a sub-selection of transcripts, and denoted any necessary additions, changes, or updates to the codes. They reviewed their coded transcripts together, assessing for consistency in code application, and discussed their suggested updates to the codebook. Upon agreeing on a finalized version of the codebook, ALK and DM coded 25% of the data, and ran an interrater reliability query to determine consistency in the application and segmentation of codes. Upon discussion and further revisions to the codebook, they recoded the previously coded text using the finalized codebook. ALK coded the remaining transcripts using the agreed upon codebook, created data reduction tables to denote frequency of major and minor codes, and drafted memos to explore relationships between themes and sub themes.

### Ethical statement

The following review boards approved the FAST study: Biomedical Research and Training Institute, the Medical Research Council of Zimbabwe, and Duke University (2019–0268). All study participants provided written informed consent.

## Results

In total, 52 young adults took part in our study (Table 2); across 10 colleges and universities. Of the total sample, four individuals had reactive self-test results which were confirmed positive with blood-based testing, and 41 had non-reactive results. One individual had a false-reactive result, which was later confirmed negative with blood-based testing. Participants cited various reasons for accessing HIVST on campus, including: recent risky sexual experience, curiosity to try a new diagnostic technology, and a desire to follow preventative health guidelines and ‘know one’s status’. Six participants opted out of campus-based HIVST, i.e., they approached the distribution team to learn more about the study, did not proceed to HIVST, but agreed to undertake a qualitative interview.

**Table 2:** Participant demographics.

	IDI	FGD
	(n = 26)	(n = 26)
*Age*, *years*		
Range	18–24	18–24
*Gender*		
Female	15	13
Male	11	13
*Place of study*		
Technical vocational college (4)	16	26
Teaching college (3)	2	–
University (3)	8	–
*HIV Self-Test Location*		
On–Site	10	20
Off–Site	10	6
Did not test	6	–
*Test Result*		
Negative	15	26
Reactive (positive)	4	–
False-reactive	1	–
*Last Tested for HIV*		
Never tested	10	–
3–12 months	4	–
13–24 months	7	–
25–60 months	5	–
*Location of previous HIV test*		
At a clinic or hospital	13	–
Self-test purchased at pharmacy	1	–
At school health clinic	1	–
At a school-based testing campaign	1	–

Of all IDI participants (n = 26), 61.5% (n = 16) reported previously testing for HIV, 81% (n = 13) of whom had accessed facility-based testing. Of participants who took a HIVST (22/26), 30% (n = 6) reported testing for the first time, with four of these first-time testers opting to test onsite. Questions related to personal history of HIV testing were not asked of FGD participants to safeguard their privacy.

Themes in the data emerged within two broad categories: acceptability of and concerns with campus-based HIVST. Factors that contributed participants’ acceptance of this distribution modality were a perceived increase in autonomy and convenience. Additionally, some participants felt that campus-based distribution of HIVST created an environment of social acceptability around HIV testing more broadly. Factors that contributed to participants’ concerns with campus-based HIVST distribution were a perceived lack of privacy and insufficient post-test counseling, as well as experiences of socially coerced testing.

### Acceptability of campus-based HIVST

The vast majority of young adults positively endorsed campus-based delivery of HIVST (19/26 IDI participants). They cited a perceived increase in autonomy, convenience, and social acceptability as attractive elements of this distribution modality.

#### Autonomy

Many participants felt that campus-based HIVST offered them more autonomy than facility or community-based HTS. Participants across study sites agreed that the flexibility of the campus-based model allowed them to determine what level of privacy they wanted, and to control who, if anyone, had access to their test results. Having the choice to decide how, when, where, and with whom to test for HIV was a highly desirable aspect of campus-based delivery of HIVST. Participants who opted to test off-site, the majority of whom reported having previously tested for HIV, noted that the ability to complete the test independently was their primary incentive for taking a HIVST.

For many, the autonomy to choose where one tested, and what support network they mobilized, was an empowering experience. One participant reflected that her experience of self-testing on campus influenced her to take a more active role in managing her sexual health through routine HIV testing:

*“But I was very happy [to take the self-test] and…let’s just say that I’m negative right now, I now know what I have to do protect myself…it’s sort of like, now a responsibility to continue to be negative and not stumble upon getting HIV. So, it’s now work to me. The experience was it was an eye-opener because I’m negative, now I have the responsibility to carry on my life in a straight manner so I don’t become a victim of HIV.” (Female, HIV-)*

#### Convenience

The majority of participants, regardless of their chosen testing location, favored the convenience of campus-based HIVST. As students, participants discussed their anxiety about maintaining academic success, while navigating new relationships and managing their sexual health. Many cited demanding exam schedules or increasing city bus fare as factors that took precedence over testing for HIV. The convenience of HIVST on campus allowed participants to better navigate their competing priorities.

Of the six participants who opted not to self-test, three reported their decision against taking a self-test on campus was in part related to pressing academic requirements which required their focus. They discussed their intent to take a self-test in the future, at a time when a potential positive test result would not interfere with their academic success:

*“I am not ready because I am facing exams, and if I found out that, that the virus–it may affect me. I think maybe after the exams when everything has settled down.” (Male, opted out of HIVST)*

#### Social acceptability

Participants who tested on-site expressed that young adult lay counselor led delivery of HIVST on campus generated an increased sense of social acceptability around HIV testing. Testing with their age cohort in a familiar environment appeared to reduce perceived stigma typically associated with testing uptake in traditional venues accessed or staffed by adults. Some participants discussed how the open layout of the distribution campaign station projected an image of student buy-in, which influenced individuals to join the crowd and take a self-test. This participant who learned of her positive status while self-testing reflected on how campus-based delivery of HIVST created an environment conducive to testing:

*“Self-testing at college, everyone was doing it…at Bindura university everyone was doing it. There is no one who will go around saying I saw so and so getting a self-test kit. No, because everyone was getting them. It wasn’t something that was unique.” (Female, HIV+)*

Participants who tested off-site also remarked that the youth-friendly communication and non-medical demeanor of the young adult lay counselors encouraged them to access the HIVST services being offered.

*“I don’t know why, I can’t explain it but those people with white clothes… just going there [to the health clinic], they are serious people…So, when I came here and saw people dressing casually in ways that I can relate to, I told myself this is a good thing, this is a good opportunity, so I came to get tested.” (Male, HIV-)*

One participant who tested positive remarked that the lay counsellors’ willingness to share openly about their own experiences testing for HIV helped her to accept her status and link to care:

“*Overall*, *they [the lay counsellors] were just fine*. *They also disclosed their statuses to me and they were like*, *‘You can hardly tell*! *That is why you probably didn’t suspect anything*, *and the reason why you can’t*, *is that we are on medication*.*’ So*, *it became easier to accept*.” *(Female*, *HIV+)*

### Concerns with campus-based HIVST

A small number of young adults (7/26 IDI participants) did not endorse campus-based HIVST, citing perceived risks associated with this distribution modality including lack of privacy, social coercion, and insufficient post-test counseling.

#### Lack of privacy

The majority of participants who tested off-site reported that the social nature of campus-based testing contributed to their decision to test at home, or in their hostel rooms; they were concerned about maintaining the privacy of their test results in front of their fellow students. While they reported the lay counselor delivery of HIVST contributed to a less stigmatizing experience of seeking testing, they preferred to test themselves privately.

Participants who opted not to test also cited lack of privacy as a concern which contributed to their decision against using campus-based HIVST. When asked about his perceptions of the campus-based HIVST distribution model, this participant shared:

*“Ah*, *I think to some extent*, *it’s of great importance*. *And*, *to a lesser extent*, *it is not kind of good …In terms of atmosphere*, *it should be comfortable*, *so that your activity is yours alone*. *Yeah*, *the atmosphere is friendly*. *But this current set-up*, *I dislike it because it is an open environment… there is transparency*. *It is too open*.*”* (*Male*, *opted out of HIVST*)

#### Social coercion

Social coercion emerged as a cross-cutting theme related to participant experiences with campus-based HIVST. The first commonly discussed type of coercion, involving young women pressuring their male partners to test, was framed as well-intentioned and empowering. Young women indicated that campus-based HIVST empowered them to promote testing and negotiate safer sex practices with their male partners, because it offered an immediate, convenient, and accessible option that they would not be able to refuse.

When asked if she thought her partner would also use the HIVST on campus, this young woman shared her perspective on how campus-based delivery of HIVST, or a secondarily distributed kit, could increase her negotiating power within her relationship:

*“I will have to force him to do it. The thing is that, if he refuses to get tested I’ll have to force him, because a self-test is only him and me. So, there’s no need for him to refuse, because no one will be watching him getting tested. So, it should be fine.” (Female, HIV-)*

The second commonly discussed type of coercion occurred between groups of friends. Given the social nature of campus-based distribution, most participants were encouraged to test by their friends, approached the distribution campaign station in groups or pairs, and often decided to test together regardless if they tested on-site or off-site. This participant who tested reactive and was later confirmed HIV positive shares that while they were originally motivated to test from the encouragement of their friends, they felt pressured to disclose their results to their friends who tested with them.

*“After getting this self-test kit, my friend came to me in the morning and she said, “I tested and I think you should do it.” Then I am like, “I don’t think I am ready yet.” I said this probably because I wanted to do it later on, I was going to save it. Then she was like, “No, just do it…just close the doors and make sure that no one is around.” So, then I did it.” (Female, HIV+)*

#### Post-test counseling and linkage to care

Participant preferences for campus-based HIVST revealed competing desires for complete autonomy while testing, and readily available access to post-test counseling services in the event of a reactive test result. Participants raised concerns about adequate access to post-test counseling services when using a HIVST on campus, citing fears that people who receive reactive results while testing alone with have adverse emotional reactions and will experience challenges in linking to care.

The perception of inadequate post-test counseling was a motivating factor for those participants opting out of campus-based delivery of HIVST. This participant discusses their preference for their partner to take a self-test at a clinic, where they anticipate she will be able to have the private, autonomous experience of self-testing while remaining safely embedded within the support network of a health facility.

*“Well, I would like her to do a self-test at a clinic if she’s comfortable to do it alone. I’m considering that maybe she might not like the results and she might decide to do a suicide of some sort…I’ve heard so many cases of when people come out positively and think that’s the end of it. But if you are around qualified people who can tell you that it is not the end of life, people who encourage you to take the medication that they give you that help maintain your healthy body, I would prefer for that person to go to a clinic and do the self-test there.” (Male, opted out of HIVST)*

Interestingly, participants with reactive test results were satisfied with the level of social support and post-test counseling offered by the young adult lay counselor team. Three of the four participants who reported testing positive with campus-based HIVST discussed how having a reactive self-test result compelled them seek confirmatory testing at a local clinic of their choosing. While they appreciated the option for additional linkage to care support, these individuals reflected on how the immediate need to confirm their results was the primary motivating factor for them to seek further testing at a clinic.

### Recommendations for future campus-based HIVST implementation

Participants offered various recommendations for improving future campus-based HIVST implementation, including increasing post-test counseling options for testers, using young adult lay counsellors from outside of the school community to distribute HIVST, and providing secondary HIVST for partner-referred testing (see S2 Table).

## Discussion

The results of our study indicate that campus-based delivery of HIVST is highly acceptable among tertiary-level students. Students favored this distribution model because it enabled them to circumvent aspects of facility-based testing they viewed as barriers, including lack of privacy, confidentiality, convenience, and autonomy. Participants who tested on-site were primarily first-time testers and favored the ‘supported, but autonomous’ experience of testing in or near the campus-based distribution booths. The majority of participants who tested off-site reported previously testing for HIV, and were comfortable taking their kit to another location for use. The flexibility of campus-based distribution enabled multiple forms of engagement, empowering participants to assess their individual needs while deciding when, where, and with whom to test. Taking charge of one’s own health motivates young adults to test for HIV [12, 27]; this resonates with our findings that a youth-friendly campus-based HIVST delivery option enabled participants to more easily access testing.

However, while many participants benefitted from the flexibility of campus-based distribution of HIVST, there were some participants who did not feel the distribution model provided sufficient privacy to comfortably test themselves for HIVST. Several participants who opted not to HIVST reported that the act of approaching the campus distribution booth in front of their peers diminished their privacy. Interestingly, this contrasts with the experience of other participants who felt that testing amongst their peers in a familiar environment generated a sense of social acceptability, projected the image of broad student buy-in, and reduced their experience of anticipated stigma. More research is needed to understand the nuances in perceptions of privacy and HIV testing for young adults.

Despite the positive reception of campus-based HIVST, participants raised several concerns. First, there were concerns about socially coerced testing. Given the social nature of the distribution sites, students approached the recruitment table in large or small groups. Often, participants decided to test on-site but did not want to wait in line to use the private testing booths, and opted to conduct their test on the grounds near the distribution campaign station. In cases such as these, several participants reported feeling subtly coerced by their friends to disclose their status after completing their self-test. It is important to note that coercive or mandatory HIV testing is never advised, but can occur inadvertently, including with HIVST [28]. It is imperative that non-facility based HIVST delivery models, including campus-based testing, develop clear protocols and communication materials so lay distributors can effectively sensitize self-testers against coercing partners, friends, or other social contacts into using HIVST.

Other notable concerns raised by participants included the lack of in-person support with campus-delivered HIVST, particularly for those who tested alone or received reactive results. Participants’ perceptions around the value of privacy while testing ranged from the desire for total privacy with no support from staff, to partial privacy with easily accessible in-person support close on hand. Participants who opted to test on-site reported that their decision was motivated by a fear of conducting the self-test improperly, and the anxiety that testing positive while alone could lead to suicide or other self-harm. The HIV Self-Testing Africa (STAR) trial found no evidence of suicides or deaths related to uptake of HIVST across six HIVST implementation studies conducted in Malawi from 2011 to 2017 [9, 29]; however, the fear of suicidal ideation associated with testing alone is consistent with findings from previous research on HIVST acceptability in both adults and young adults [30–32]. Both on-site and off-site testers recommended increasing in-person support for first-time testers, and expanding post-test counselling options for couples in newly serodiscordant relationships and those with reactive test results.

Despite varying levels of need for staff support while testing, participants agreed that HIVST distributors should be young adult lay counselors, preferably from outside the school community. This finding differs from recent studies among young adults in Nigeria and Uganda that showed participants favored peer-to-peer distribution of HIVST, where they could access self-tests from a trusted leader of a social network within their community such as a savings group or girls club [33, 34]. This difference could be due to the highly networked nature of student communities within schools. A young adult counselor who is relatable, but not part of the school community, may be an attractive feature for participants wanting to easily access testing while maintaining privacy.

Participants also recommended that future campus-based HIVST delivery offer secondary distribution kits for partners, friends, or family members. Female participants in particular discussed how partner-delivery of HIVST would be a valuable extension to campus-based programming, as it would offer them an empowering tool to negotiate testing with their male partners. In 2019, the WHO recommended social-network based testing as part of an updated package of partner services among key populations [35]; index testing and secondary HIVST kit distribution were among novel testing approaches cited for consideration within these communities. While early evidence suggests they may increase HIV diagnoses, identify additional PLWHV, and increase acceptability of partner HIV services among key populations [35, 36], there is limited research that examines the efficacy of these modalities among young adults. A recent study examining social harms incurred from partner-delivered HIVST found that coercion to test between adult partners in established relationships was seen as well-intentioned and socially appropriate [9]; however, currently it is unclear if coercion to test would be similarly interpreted during partner-delivered HIVST between young adults. More research is required to understand the acceptability and possible risks of these novel recruitment methods among young adults, as well as the feasibility of incorporating these methods into campus-based HIVST distribution.

This study is not without its limitations. The study focused primarily on participants attending technical vocational colleges, the majority of which were located in Harare. This may reduce the breadth of our findings. In addition, our relatively small sample size and non-randomized sampling methods reduces the generalizability of the study. However, our findings are consistent with those from two campus-based HIVST distribution studies in South Africa and Democratic Republic of Congo [18, 19], and they further contribute to our understanding of young adults’ acceptability and preferences of campus-based, youth-friendly HIVST distribution.

## Conclusions

As more PLWHV become aware of their status, innovative testing strategies are required to reach the first of the UNAIDS 95-95-95 goals by 2030. As a screening tool, HIVST is consistent with PEPFAR recommendations for scaling up efficient strategies that test fewer people but identify higher numbers of positive cases [37]; however, HIVST distribution methods need to be strategically designed to reach distinct subpopulations. Our findings indicate that campus-based delivery of HIVST at tertiary-level schools in Zimbabwe is highly acceptable among young adults, and that uptake of HIVST through this distribution model is most promising if young adult lay counselors distribute self-tests, continuous counseling options are improved, and information sensitizing young people about socially coerced testing is provided alongside testing services. Implementing campus-based HIVST, alongside a combination of targeted HIVST strategies such as facility, community, and social network-based testing approaches will likely be necessary to close the gap in diagnostic coverage for young adults in Zimbabwe.

## Supporting information

S1 File COREQ checklist. (DOCX)

S2 File IDI topic guides. (DOCX)

S3 File FGD topic guide. (DOCX)

S1 Table Study site demographics. (XLSX)

S2 Table Implementation recommendations for campus-based HIVST distribution. (XLSX)
