# Supplementary material for: Youth-friendly HIV self-testing: Acceptability of campus-based oral HIV self-testing among young adult students in Zimbabwe
Source: PLoS One. 2021 Jun 29;16(6):e0253745. doi: 10.1371/journal.pone.0253745 (PMC8241036; doi:10.1371/journal.pone.0253745)
Supplement: S2 File — (DOCX) [file pone.0253745.s002.docx]

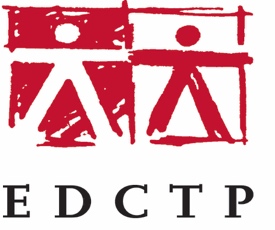
**
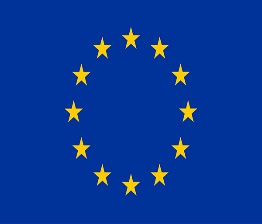
**

**FAST In-Depth Interview Topic Guide**

**(Feasibility and acceptability of HIV self-testing in adolescents and young people)**

**Reminder to RAs:**

The overall purpose of the in-depth interview is to understand:

- the feasibility and acceptability of delivering university or college campus-based HIV self-testing services to young people
- adolescents’ and young peoples’ access to, uptake of, and recommendations for these services
- the barriers or facilitators of linkage to care in this HIV self-testing intervention

***The ultimate goal of the data collection is to explore participants’ perceptions of the HIVST,***

***preferences about the FAST distribution process, and experiences with linkage to care.***

The questions included here are not exhaustive and they are not prescriptive. This means that as the interview progresses, you may ask questions that are not included below, and similarly, it may not be appropriate or necessary to ask all of the questions included in this topic guide- *the discussion should be guided by what your participant says, NOT by the topic guide.* For that to happen, you should make sure that you’re familiar with the guide so that so that you can engage more fully in the discussion, and be responsive to what the participant is telling you, by exploring these responses further. *Try to integrate some of the information that they have told you into your subsequent questions – this will demonstrate that you’re listening, and give participants a chance to clarify anything you might be misunderstanding*. It is important for you to show that you are interested in what they are saying, and that you are there to learn from them.

**Introduction to Participant**

***(note: this is an example so that you can see how we’d like the interview and the process to be explained, but you will likely want to adapt this to your own style)***

Thank you for taking the time to speak with me today.

My name is _______________ and I am working with the FAST research project to learn more about your experience with HIV self-testing. I would like to talk to you about your own experiences, ideas and opinions regarding HIV-testing for adolescents and young people. Specifically, I would like to learn about your experience using the HIV self-test, and about how access to and use of this type of service can be improved.

The interview will take about 60 minutes. I appreciate you spending this time with me.

I am going to audio record the interview to make sure that I capture all the valuable information that you share with me. I may also write things down while we’re talking so that I don’t forget anything. Participation is voluntary- you do not have to answer any question that you don’t want to, and you can choose to stop the interview at any time.

Everything you say is confidential, so please feel free to talk about your experiences and ideas. We will not record your name anywhere, and no one else will hear the tape or see the notes besides the people who are working on this research project. We may use some of what you say in reports or publications, but will never use your name.

If you have any questions about this study, you can ask me now, or at any time during our conversation ***(RA: make sure you have collected signed consent form and answered any questions the participant has.)***

Start the ***tape recorder***.

**Participant: HIV -**

| Unique Participant ID: ________________­­____ Name of Interviewer: ____________________________ Date (Day/Month/Year): ____________________  *Please mark*:  1^st^ Interview  Follow-up Interview *Please mark*:  Female  Male Study Site: _______________________________ |
| --- |

| **Topic** | **Rationale** | **Suggested Questions** |
| --- | --- | --- |
| **I. Getting to know the participant** | Initially we want the respondent to feel comfortable talking to us. These questions are meant to ease them into discussion. | 1. **Tell me about how you’ve been spending your time this week:**    1. What course are you in? How are classes going?    2. What do like to do outside of class?    3. Do you live in the hostels, or somewhere else?    4. What do you and your friends like to do?    5. Do you have a boyfriend or girlfriend/spouse? |
| **II. Participant experience of HIVST** | In this section we want to understand the participant’s experience of using an HIVST through the campus-based distribution program. | 1. **What made you decide to approach the HIVST distribution station on campus?**   *[NOTE: What made the booth a safe space for young people to approach?]*   1. **Why did you decide to pick up an HIVST from the on-campus station?** 2. Why did you opt for a HIVST, instead of testing at a clinic? 3. *If the respondent preferred self-testing to clinic testing:* What is it about HIV self-testing that felt preferable to testing at a clinic? 4. **Tell me a bit about what your experience with the HIVST was like.** 5. Where did you take your HIVST (*i.e. in the testing booth, on the grounds, at home*) and why? 6. Did you feel comfortable taking your test there? Why or why not? 7. Were you alone when you took your HIVST? 8. If you were alone, how did you feel taking the test by yourself? Did you want anyone else there with you? 9. Did you find it easy or difficult to read the results of your test? 10. Was it possible for you to get help from the staff at the distribution site? 11. Was there anything you disliked about the process (i.e. the queue, etc.)? 12. *If the participant has had a blood-based HIV test in the past****:* What was different about your experience with the HIVST, compared to your experience with a blood-based test?** 13. **Do you plan to test for HIV in the future? Do you think you will use a self-test or test at the clinic? Why or why not?** |
| *I know that throughout this process you have learned that you are HIV negative. I’d like to spend some time talking about how your experience has*  *been learning about your status through a HIVST. I hope you’ll feel comfortable talking to me about this.*  NOTE: If participant says they tested HIV+, switch to the HIV + participant interview guide and begin from Section III. | | |
| **III. Disclosure and partner referral** | In this section we want to learn about how this HIV self-testing intervention facilitated disclosure and/or partner referral. | 1. **Did you tell any of your friends, family members, or partner that you decided to take a HIVST? Why or why not?** 2. *If participant has stated they have a boyfriend or girlfriend:* **Did you request that your partner take an HIVST? Why or why not?** 3. Have you ever asked your partner to take a HIV test before? Did they test, why or why not? 4. **If the distribution program gave you a test kit to give to your partner or partners, do you think they would use it?** 5. Would you feel comfortable giving a HIVST to your partner or partners? 6. If you wanted your partner or partners to test for HIV, would it be easier to convince them to use a HIV self-test, or get a test at the clinic? |
| **IV. Support while HIV self-testing** | In this section we are trying to understand how the mental health and emotional response of participants who used a HIVST.  We want to understand if participants feel that they had adequate support from the distribution staff. | 1. **Before you found out the results of your test, did you feel that you could ask for help or support from the HIV self-testing team?** 2. Did you feel you could use the call-me-back service, seek face-to-face help from a member of the study team, and/or use the clinic referrals if you needed to link to care? 3. **What, if any, additional counseling or support would you have liked before, during, or after you took the HIV self-test?** 4. **We’ve heard from lots of young people that they were nervous to take an HIVST alone because of different fears they have.**   a. Why do you think young people have these fears when using a HIVST?  b. Did YOU yourself have any fears when you were deciding to take the test? |
| *We’ve just about come to the end of our interview. I’d like to finish up by asking a few more questions, this time about your thoughts and ideas about how we can make the HIV self-testing experience better for all people, but especially for youth.* | | |
| **V. Recommendations for future implementation** | In this section we would like to learn about individual opinions about how to improve future implementation of campus-based delivery of HIVST. | 1. **For young people who test positive with an HIVST accessed on campus, what is the most important thing they need after they get their diagnosis?** 2. Support to overcome initial shock, sadness, and fear 3. Support to reduce shame and help with partner disclosure 4. Support to link to care and start ART 5. **What are some of the barriers to delivering HIVST to young people at colleges or universities?**   a. What can be done to overcome these barriers?   1. **If you were designing a program for providing HIVST on campus for young people, what would it look like, or what do you think it should definitely contain?**   a. Programs that let you take HIV self-test kits home to family, partners, or friends |
| **VI. Wrapping up** | This is to ensure that we haven’t missed anything, but also to help people revisit points that they would expand on now since they have been thinking about it throughout the interview process.  It also leaves people feeling that they have made a valuable contribution. | 1. **Thank you so much for sharing your time with me. I appreciate you being open with me, and offering your time to answer my questions. Have I neglected to ask any questions you feel are important to understanding your experience with the HIV self-test?** 2. **Do you have any questions for me?** 3. **May I contact you by phone in the future if I need to ask any follow-up questions?** |

**Participant: HIV +**

| Unique Participant ID: ________________­­____ Name of Interviewer: ____________________________ Date (Day/Month/Year): ____________________  *Please mark*:  1^st^ Interview  Follow-up Interview *Please mark*:  Female  Male Study Site: ______________________________ |
| --- |

| **Topics** | **Rationale** | **Suggested Questions** |
| --- | --- | --- |
| **I. Getting to know the participant** | Initially we want the respondent to feel comfortable talking to us- these questions are meant to ease them into the discussion, before we discuss the result of their recent HIVST. | 1. **Tell me about how you’ve been spending your time this week:**    1. What course are you in? How are classes going?    2. What do like to do outside of class?    3. Do you live in the hostels, or somewhere else?    4. What do you and your friends like to do?    5. Do you have a boyfriend or girlfriend/spouse? |
| *I know that throughout this process you have learned that you are HIV positive. I’d like to spend some time talking about how your experience has*  *been learning about your status through a HIVST. I hope you’ll feel comfortable talking to me about this.*  NOTE: If participant says they tested HIV-, switch to the HIV - participant interview guide and begin from this section | | |
| **II. Participant experience of HIVST** | In this section we want to understand the participant’s experience of using an HIVST, and the campus-based distribution program. | 1. **Why did you decide to pick up an HIVST from the on-campus station?** 2. Why did you opt for a self-test, instead of testing at a clinic? 3. *If the respondent preferred self-testing to clinic testing:* What is it about HIV self-testing that felt preferable to testing at a clinic? 4. **Tell me a bit about what your experience with the HIVST was like.** 5. Where did you take your HIVST (i.e. in the booth, on the campus grounds, off-site?) 6. Did you feel comfortable taking your test there? Why or why not? 7. Were you alone when you took your HIVST? 8. If you were alone, how did you feel taking the test by yourself? Did you want anyone else there with you? 9. Did you find it easy or difficult to read the results of your test? 10. Was it possible for you to get help from the staff at the distribution site? 11. Was there anything you disliked about the process (i.e. the queue, etc.)? 12. *If the participant has had a blood-based HIV test in the past****:* What was different about your experience with the HIV self-test, compared to your experience with a blood-based test?** |
| **III. Linkage to care** | We are interested to learn if the linkage to care support offered through this young adult lay counsellor led campus-based HIVST program is acceptable and sufficient for AYP who test positive with a HIVST accessed on campus. | 1. **After you read the result of your HIVST, did you seek support from the lay counselors, or from a trusted friend or family member, to figure out what steps to take next?** 2. *If participant says they have not yet linked to care:* **What kind of support do you think you need to go to a clinic and begin linking to care?** 3. *If student has not linked to care, skip this question. If they have:* **Do you feel ready to start ART? Why or why not?** 4. **What are the challenges of linking to care when you test positive with a HIVST you picked up on your college campus?** |
| **IV. Disclosure and partner referral** | In this section we want to learn about how this HIVST intervention facilitated disclosure and/or partner referral. | 1. **Did you tell any of your friends, family members, or partner that you decided to take a HIVST? Why or why not?** 2. *If participant has stated they have a boyfriend or girlfriend:* **When you went to get an HIVST, did you also request that your partner take an HIVST? Why or why not?** 3. Have you ever asked your partner to take a HIV test before? Did they test, why or why not? 4. **Have you shared your test result with any family, friends, or a partner?** 5. **If the distribution program gave you a test kit to give to your partner or partners, do you think they would use it?** 6. Would you feel comfortable giving a HIV self-test to your partner or partners? 7. If you wanted your partner or partners to test for HIV, would it be easier to convince them to use a HIV self-test, or get a test at the clinic? Why? 8. **Do you think campus-based HIVST programs should offer provide support to couples navigating status disclosure?** |
| **V. Support while HIV self-testing** | In this section we want to understand if participants who tested positive using an HIVST accessed on campus feel that they had adequate support from the young adult lay counsellor staff.  We want to learn about their thoughts and opinions about how a campus-based HIVST intervention could be improved to better support those who test positive. | 1. **Did you feel that you could seek help from the lay counselors at the HIVST distribution station, or via another method?** 2. Did the participant feel they could use the call-me-back service, seek face-to-face help from a member of the study team, and/or use the clinic referrals for linking to care? 3. *If self-tested alone:* **Do you think that testing for HIV by yourself affected your emotional response to your HIV diagnosis?** 4. If so, why? 5. *If self-tested with someone*: **Do you think that using an HIVST that you picked up on campus with** [person they tested with] **affected your emotional response to your HIV diagnosis?** 6. **Have you come to accept your diagnosis? Why or why not?**    1. *If accepted:* What do you think helped you to become more at peace with your HIV diagnosis? (support received, realizations, disclosures, etc.)    2. *If not yet accepted:* What kinds of support would have helped you in this process? (partner, family, community, health providers/clinic support, professionals, etc.) 7. **What, if any, additional counseling or support do you feel that you need?** |
| **VI. Recommendations for future implementation** | In this section we would like to learn about individual opinions about how to improve future implementation of campus-based delivery of HIVST. | 1. **For young people who test positive with an HIVST accessed on campus, what is the most important thing they need after they get their diagnosis?** 2. Support to overcome initial shock, sadness, and fear 3. Support to reduce shame and help with partner disclosure 4. Support to link to care and start ART 5. **What are some of the barriers to delivering HIVST to young people at colleges or universities?** 6. Is there anything that could be done to overcome these barriers? 7. **If you were designing a program for providing HIVST on campus for young people, what would it look like, or what do you think it should definitely contain?**   a. Programs that let you take HIV self-test kits home to family, partners, or friends |
| **VII. Wrapping up** | This is to ensure that we haven’t missed anything, but also to help people revisit points that they would expand on now since they have been thinking about it throughout the interview process.  It also leaves people feeling that they have made a valuable contribution. | 1. **Thank you so much for sharing your time with me. I appreciate you being open with me, and offering your time to answer my questions. Have I neglected to ask any questions you feel are important to understanding your experience with the HIV self-test?** 2. **Do you have any questions for me?** 3. **May I contact you by phone in the future if I need to ask any follow-up questions?** |

**FAST In-Depth Interview Topic Guide**

**(Feasibility and acceptability of HIV self-testing in adolescents and young people)**

**Participant: Opted Against HIVST**

| Unique Participant ID: ___**N/A**_______________ Name of Interviewer: ____________________________ Date (Day/Month/Year): ____________________  *Please mark*:  1^st^ Interview  Follow-up Interview *Please mark*:  Female  Male Study Site: _____________________________ |
| --- |

| **General Topics** | **Rationale** | **Suggested Questions** | |
| --- | --- | --- | --- |
| **I. Getting to participant** | Initially we want the respondent to feel comfortable talking to us- these questions are meant to ease them into discussion. | 1. **Tell me about how you’ve been spending your time this week:**    1. What course are you in? How are classes going?    2. What do like to do outside of class?    3. Do you live in the hostels, or somewhere else?    4. What do you and your friends like to do?    5. Do you have a boyfriend or girlfriend/spouse? | |
| **II. Participant perceptions of HIV self-testing on campus** | In this section we want to understand the participant’s perceptions of the campus-based HIVST program, and the reasons why they opted against HIVST. | 1. **To start off, before we set up our distribution booths here on campus, had you ever heard of, or used an HIV self-test before coming to our booth?** 2. Where did you hear about it? What did you hear about it? 3. **What made you decide to approach the booth to learn more about what service was being offered?** 4. **We’ve heard from lots of young people that they were nervous to take an HIV self-test alone because of different fears. Did you have any fears when you were deciding to take the test?**   a. What fears were those?   1. **Do you want to test for HIV? Why or why not?** | |
|  |  | *If Yes*   1. **Why did you decide not to take the HIVST?** 2. Was there anything about the distribution station that affected your decision not to test?   *[NOTE: Did the participant feel comfortable getting an HIVST in that space?]*   1. Did you feel your anonymity would be compromised if you used an HIVST from the test booth?   *[NOTE: Did the participant’s anticipation of stigma influence them against testing with the self-test option?]*   1. **Do any of your friends or family (or partner) know that you wanted to take a HIV self-test? Why or why not?** 2. **What do you think are some of the reasons that other young people might decide not to test for HIV with an HIV self-test?** 3. **Do you plan to get tested in the future?**   *If yes:* What kind of test will you use, and why? | *If No*   1. **Tell me more about why you did not want to test for HIV.** 2. Did you feel your anonymity would be compromised if you used an HIV self-test from the test booth? 3. Was there anything about the self-test distribution booth that affected your decision not to test? 4. **What do you think are some of the reasons that other young people might decide not to test for HIV with an HIV self-test?** 5. **Do you plan to get tested in the future?**   *If yes:* What kind of test will you use, and why? |
| **III. Recommendations for future implementation** | In this section we would like to learn about individual opinions about how to improve future implementation of campus-based delivery of HIVST. | 1. **What would you recommend that the team change about the distribution booth so that young people would feel comfortable approaching and getting a test?** 2. **If you were designing a program for providing HIVST on campus for young people, what would it look like, or what do you think it should definitely contain?**   a. Programs that let you take HIV self-test kits home to family, partners, or friends   1. **What is the most important message we can provide for youth when they are testing for HIV for the first time, using an HIV self-test?** | |
| **IV. Wrapping up** | This is to ensure that we haven’t missed anything, but also to help people revisit points that they would expand on now since they have been thinking about it throughout the interview process. | 1. **Have I neglected to ask any questions you feel are important to understanding your experience with the HIV self-test distribution program?** 2. **Do you have any questions for me?** | |
