# Supplementary material for: Youth-friendly HIV self-testing: Acceptability of campus-based oral HIV self-testing among young adult students in Zimbabwe
Source: PLoS One. 2021 Jun 29;16(6):e0253745. doi: 10.1371/journal.pone.0253745 (PMC8241036; doi:10.1371/journal.pone.0253745)
Supplement: S3 File — (DOCX) [file pone.0253745.s003.docx]

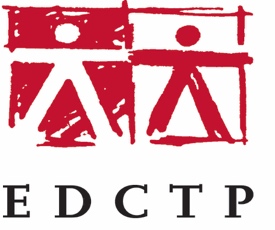
**
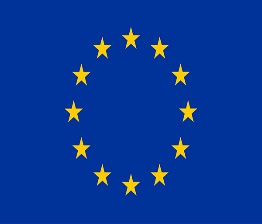
**

**FAST Focus Group Discussion Guide**

**(Feasibility and acceptability of HIV self-testing in adolescents and young people)**

**Focus Group Discussion Participants**

| Study Site: __________________________ Name of Interviewers: ____________________________ Date (Day/Month/Year): ____________________ |
| --- |

**Reminder to RAs:**

The overall purpose of the in-depth interview is to understand:

- the feasibility and acceptability of delivering university or college campus-based HIV self-testing services to young people
- adolescents’ and young peoples’ access to and uptake of these services
- the barriers or facilitators of linkage to care in this HIV self-testing intervention

***The ultimate goal of the data collection is to explore participants’ perceptions of the HIV self-test,***

***preferences about the FAST distribution process, and experiences with linkage to care.***

The questions included here are not exhaustive and they are not prescriptive. This means that as the focus group progresses, you may ask questions that are not included below, and similarly, it may not be appropriate or necessary to ask all of the questions included in this topic guide- *the discussion should be guided by what your participants say, NOT by the topic guide.* For that to happen, you should make sure that you’re familiar with the guide so that so that you can engage more fully in the discussion, and be responsive to what the participant is telling you, by exploring these responses further. *Try to integrate some of the information that they have told you into your subsequent questions – this will demonstrate that you’re listening, and give participants a chance to clarify anything you might be misunderstanding*. It is important for you to show that you are interested in what they are saying, and that you are there to learn from them.

**Introduction to Participants**

***(note: this is an example so that you can see how we’d like the interview and the process to be explained, but you will likely want to adapt this to your own style)***

Thank you for taking the time to speak with us today.

My name is _______________, and I am working with the FAST research project to learn more about how young people like yourselves view HIV self-testing. This discussion will take between one and one and a half hours. Before we start on the questions, we would like to all agree on small rules that will help us have a fruitful discussion – the first is that we would like everyone to participate and listen to each other’s opinions. The second is that we would prefer, as much as possible, if one person spoke at a time so we can accurately hear everyone. The third, is that everything you say in this group is confidential, so please feel free to talk about your experiences and ideas. We will not record your name anywhere, and no one else will hear the tape or see the notes besides the people who are working on this research project. We may use some of what you say in reports or publications, but will never use your name. We ask all of you to keep the confidence of the other members of the group, and commit to not repeating anything that anyone says while we are in this discussion circle.

I am going to audio record the interview to make sure that I capture all the valuable information that you all share with me. I may also write things down while we’re talking so that I don’t forget anything. Participation is voluntary- you do not have to answer any questions that you don’t want to, and you can choose to leave the group at any time.

If you have any questions about this study, you can ask me now, or at any time during our conversation ***(RA: make sure you have collected signed consent form and answered any questions.)***

Start the ***tape recorder***.

| **Topic** | **Rationale** | **Suggested Questions** |
| --- | --- | --- |
| **I. Getting to know participants, assigning code names** |  | **Let’s begin by assigning everyone with a code number. Please use this number to refer to yourself, and refrain from using your name or names of friends during this recorded conversation. For example, when you want to share your opinion, you can raise your hand and begin by saying, “Number 12.” and then proceed.** |
| **II. Opinions and preferences for HIV self-testing vs. HIV testing in the clinic** | *We will begin by probing about people’s preferences about whether or not they prefer to use a self-test, or test in the clinic.* | **1.** **Let’s think back to when you decided to take the HIV self-test. What made YOU say to yourself, “I want to take this test?”**   - Why did you decide to take a self-test, instead of testing at a clinic? - For those who have taken a blood-based HIV test before, what was different about your experience with the HIV self-test, compared to your experience with the blood-based test? |
| **III. Opinions and preferences for testing on-site or off-site** | *In this section we want to explore participant choice around where the chose to test, and why.* | **2.** **Did any of you take the self-test on-site? Tell me about why you chose to test on-site.**   - Why did that feel more comfortable for you than testing by yourself, off-site? - Did you receive help or support from the research team? - Was that [receiving help from the study team] an important factor in making you feel comfortable to take a self-test?   **3.** **Did any of you take the self-test off-site? Tell me about why you chose to test off-site.**   - Why did that feel more comfortable for you than testing by yourself, on-site? - Was the ability to test by yourself an important factor in making you feel comfortable to take the self-test?   **4.** **It was really interesting that lots of your peers said they preferred the HIV self-test to testing in a clinic, because it was more private and ‘no one would know what they were doing’, but lots of people who wanted to test on-site decided to take the test while sitting on the grounds, in front of lots of people. Why do you think this felt more private to them than testing in a clinic?**   - Do you feel more stigma when you think people your age are watching you stand in a queue to get a HIVST, or more stigma when you think people of different ages are watching you stand in a queue to get and HIV test? |
| **IV. Recommendations for future implementation of HIV self-testing on campus** | *In this section we want to learn about participant’s recommendations for improving future campus programs for distributing HIVST. Begin by asking broad questions about their recommendations, and follow-up on commonly held recommendations.* | **5.** **What recommendations do you have to improve campus-based delivery of HIV self-tests, for students of your age?**  **6. Throughout the distribution campaign, many people expressed concern that doing an HIV self-test alone was frightening to them, that it might lead them to suicide. What could we do in future campus-based HIVST campaigns to ensure people are not afraid of this?**   - What do students need, to not fear using an HIVST alone when picking up a test kit on campus? - How could we improve our campaign in the future to make less people fear testing alone?   **7. Think back to how our campaign is structured. What would you recommend we change, to improve our support of people who test positive?**  **8. Is it important that a person find out about their HIV self-test result with another person, like a nurse, lay counselor, or a trusted friend or partner? Why or why not?**   - Would you recommend we offer supervised HIVST in private booths, alongside the option of unsupervised HIVST in private booths, and testing off-site? |
